# Diurnal oscillation of CSF Aβ and other AD biomarkers

**DOI:** 10.1186/s13024-017-0161-4

**Published:** 2017-05-08

**Authors:** Brendan P. Lucey, Anne M. Fagan, David M. Holtzman, John C. Morris, Randall J. Bateman

**Affiliations:** Department of Neurology, Washington University School of Medicine, 660 South Euclid Avenue, Campus Box 8111, St Louis, MO 63110 USA

**Keywords:** Alzheimer’s disease, Amyloid-β, Biomarkers

## Abstract

To assess stages of Alzheimer's disease (AD) pathogenesis and the efficacy of drugs during clinical trials, there has been immense interest in the field to establish baseline cerebrospinal fluid (CSF) concentrations for potential AD biomarkers such as amyloid-β (Aβ) and tau. Significant within-person variations in CSF Aβ concentrations over time found that this variation followed the sleep-wake cycle. A recent paper in Molecular Neurodegeneration reported the absence of diurnal variations in multiple classical and candidate AD biomarkers, such as soluble APP, Aβ, tau, p-tau, YKL-40, VILIP-1, or apolipoprotein E. This commentary addresses these apparently discordant results regarding the diurnal variability of APP and Aβ compared with the literature. Despite our concerns, we appreciate the authors' interest in this important topic and contribution to improve our knowledge about the factors influencing Aβ diurnal variation.

## Background

To assess stages of Alzheimer’s disease (AD) pathogenesis and the efficacy of drugs during clinical trials, there has been immense interest in the field to establish baseline cerebrospinal fluid (CSF) concentrations for potential AD biomarkers such as amyloid-β (Aβ) and tau. For instance, lumbar punctures performed in individuals demonstrate low levels of Aβ42 in individuals cerebral with amyloid deposition [1]. Large fluctuations in Aβ levels also were noted in individuals over years when sampled by lumbar puncture [2]. Indwelling lumbar catheters have also been used to obtain more frequent CSF sampling over 24–48 h to understand Aβ metabolism for pharmacological modeling during drug trials. These catheter studies found significant within-person variability in CSF Aβ concentrations over the sampled time [3].

After Aβ release was demonstrated to be driven by neuronal activity in vivo [4], further investigations in rodents and humans found that the central nervous system concentration of Aβ fluctuated with the sleep-wake cycle. The amplitude of Aβ oscillation was decreased by both amyloid deposition in the brains of AD mouse models and humans, and in individuals with autosomal dominant AD mutations [5–7]. Soluble amyloid precursor protein (APP) and plasma Aβ also oscillate with sleep-wake activity [8, 9]. An analysis of serial Aβ concentrations from 178 participants who underwent CSF sampling via indwelling lumbar catheters in multiple academic and industry studies found an Aβ diurnal pattern in 16 of 17 studies [10]. The only study without a diurnal oscillation was confounded by a bacterial filter placed on the catheter during collection that removed Aβ from the CSF samples [11]. The Aβ diurnal pattern has also been replicated using different assays (enzyme-linked immunosorbent assay and mass spectrometry) [12].

## Main text

Recently, Cicognola et al. [13] reported the absence of diurnal variations in multiple classical and candidate AD biomarkers, such as soluble APP, Aβ, tau, p-tau, YKL-40, VILIP-1, or apolipoprotein E. The authors sampled from 13 neurosurgical patients by either ventricular or lumbar CSF drainage at six time points over a 24-h period: 08:00, 12:00, 16:00, 20:00, 00:00, and 08:00. Four ml of CSF was collected at each time point.

Several factors may have led to the apparently discordant results regarding the diurnal variability of APP and Aβ, compared with the literature, in the Cicognola et al. study. First, it is possible that neurosurgical patients may have disrupted sleep-wake cycles that might alter diurnal patterns, particularly if the patients were sampled while in intensive care units where sleep would be fragmented due to frequent interruptions for clinical care. Further, brain tumors, hydrocephalus, and other intracranial pathologies could independently disturb sleep-wake activity and CSF flow.

Second, a longer sampling period may be necessary to observe fluctuations of Aβ in this population. For example, sampling periods up to 168 h were required to correlate Aβ fluctuations with changes in neurological status in patients with brain injury [14].

Third, the authors collected CSF from two different types of drains. Presumably, CSF was collected during the patients’ routine clinical care between the research sampling time points. Previous reports have found that more frequent sampling will increase the rate of linear rise in Aβ [10, 15]. The authors do not record how much CSF was drained between these time points for clinical purposes to control for this effect.

Fourth, we expect that CSF sampling was not frequent enough to detect an Aβ oscillation over 24 h and that the study is underpowered to detect any oscillation that may be present in this patient population.

Fifth, a repeated measures ANOVA may not be the correct test to determine if there is a diurnal oscillation as we would predict Aβ concentrations to oscillate together over the 24 h day.

## Conclusion

Understanding how Aβ concentrations change over short periods of time (e.g. hours) in the human central nervous system is critical to the design and implementation of anti-Aβ AD treatment trials. Despite our concerns, we appreciate the authors’ interest in this important topic and contribution to improve our knowledge about the factors influencing Aβ diurnal variation.

## Abbreviations

AD: Alzheimer’s disease
ANOVA: Analysis of variance
APP: Amyloid precursor protein
Aβ: Amyloid-β
CSF: Cerebrospinal fluid
