# Supplementary material for: Diurnal oscillation of CSF Aβ and other AD biomarkers
Source: Mol Neurodegener. 2017 May 8;12:36. doi: 10.1186/s13024-017-0161-4 (PMC5421331; doi:10.1186/s13024-017-0161-4)
Supplement: Related texts

# Reply: Author’s response: Is there an association of diurnal variation with cerebrospinal fluid biomarkers for Alzheimer’s disease?

**Authors:** Claudia Cicognola, Davide Chiasserini, Paolo Eusebi, Ulf Andreasson, Hugo Vanderstichele, Henrik Zetterberg, Lucilla Parnetti, Kaj Blennow

**Affiliations:** 2 0000 0000 9919 9582 grid.8761.8 Institute of Neuroscience and Physiology, Department of Psychiatry and Neurochemistry, The Sahlgrenska Academy at University of Gothenburg, Mölndal, Sweden; 3 000000009445082X grid.1649.a Clinical Neurochemistry Laboratory, Sahlgrenska University Hospital, Mölndal, SE-431 80 Mölndal, Sweden; 4 0000 0004 1757 3630 grid.9027.c Section of Neurology, Department of Medicine, Center for Memory Disturbances, University of Perugia, Sant’ Andrea delle Fratte, 06132 Perugia, Italy; 5 ADx NeuroSciences, Gent, Belgium; 6 0000000121901201 grid.83440.3b UCL Institute of Neurology, Queen Square, London, UK

We thank Lucey and co-workers [16] for their interest in our paper [13]. The authors point out some potential confounders that may influence sleep-wake cycles and variability of biomarkers for Alzheimer’s disease pathophysiology, thereby affecting the possibility to identify diurnal patterns of variation in biomarker concentrations [16]. As acknowledged in the discussion section of our paper, these types of potential confounders may underlie the inconsistencies in outcomes between studies, as also recently reviewed [17]. For example, acute brain trauma [18] and other possible disease- or drug-related effects on brain functioning and Aβ metabolism in neurosurgical patients ([13, 14] = Brody), may give different results than studies in healthy controls [10, 19]. Further, sampling procedures, e.g., the use of lumbar or ventricular catheters [10, 13, 19] or intracerebral microdialysis catheters [14], or even plasma [9], reflect different compartments that will impact the results.

Undoubtedly, we need to perform more studies using highly standardized protocols, controlling for clinical confounder as well possible artefacts due to variable sampling time-points and volume-related disturbances in CSF dynamics [15], to resolve whether Aβ and other biomarkers reflecting AD pathophysiology vary with the normal sleep-wake cycle, and show a true diurnal variation.
